# A systematic review of the epidemiology of epilepsy in Mexico during 1970 to 2020

**DOI:** 10.1055/s-0042-1758647

**Published:** 2023-03-14

**Authors:** Gerardo Quiñones Pesqueira, Daniel San-Juan, Rosana Huerta Albarrán, Maximo Leon Vazquez, Gerardo Quiñones Canales, Jorge Gonzalez Pesqueira

**Affiliations:** 1 National Institute of Neurology and Neurosurgery Manuel Velasco Suárez, Department of Neurophysiology, Mexico City, Mexico.; 2 National Institute of Neurology and Neurosurgery Manuel Velasco Suárez, Epilepsy Service, Mexico City, Mexico.; 3 Hospital General de México Dr Eduardo Liceaga, Department of Neuropediatrics, Mexico City, Mexico.; 4 Insituto Mexicano del Seguro Social Centro Médico Nacional La Raza, Department of Neuroscience, Mexico City, Mexico.; 5 Instituto de Seguridad y Servicios Sociales de los Trabajadores del Estado, Durango, Department of Neuroscience, Mexico.; 6 University of Arizona, College of Medicine-Phoenix, Phoenix, AZ, United States.

**Keywords:** Epilepsy, Epidemiology, Prevalence, Mexico, Rural Areas, Urban Area, Developing Countries, Epilepsia, Epidemiología, Prevalencia, México, Medio Rural, Area Urbana, Países en Desarrollo

## Abstract

**Background** Epilepsy is the most common major neurological disorder that affects people of all ages. The prevalence varies from one country to another and even between different areas, due to a lack of access to medical care for reasons related to limited resources.

**Objective** Epilepsy is a worldwide public health problem that affects more deeply populations living in developing countries such as Mexico, where more aggressive health policies based on epidemiological data are needed; however, this information is scarce and the evolution of this data over time remains unclear. The aim of the present study is to provide an overview of the epidemiology of epilepsy in Mexico from 1970 to 2020.

**Methods** We searched descriptive epidemiological studies on epilepsy in rural and urban regions of Mexico from 1970 to 2020. Available data on the sociodemographic characteristics, prevalence, and incidence data were extracted. Finally, the abstract, full-text review, and data abstraction were conducted in duplicate and reported using the Preferred Reporting Items for Systematic Reviews and Meta-Analyses (PRISMA) guidelines. Descriptive statistics was also used.

**Results** Overall, 11 underrepresented and heterogeneous epidemiological studies were included. In total, the prevalence of epilepsy in Mexico is 3.9 to 41:1,000 inhabitants; 3.9 to 41 per 1,000 persons in rural regions, and 3.49 to 44.3 per 1,000 persons in urban regions. None of these studies addressed the incidence of epilepsy. The prevalence of epilepsy in Mexico has remained unchanged during the last 5 decades.

**Conclusions** Our results confirm a high prevalence of epilepsy in both urban and rural settings in Mexico that remain unchanged during the last 5 decades. All studies included in the present review showed multiple methodological limitations. New and robust epidemiological studies are needed to delineate the epidemiological profile of epilepsy in Mexico.

## Resumen

**Antecedentes** La epilepsia es el trastorno neurológico más común que afecta a individuos de todas las edades. La prevalencia varía entre paises e incluso entre diferentes áreas del mismo pais debido a la falta de acceso a la atención médica.

**Objectivo** La epilpesía es un problema de salud pública mundial que afecta de manera más importante a los países en desarrollo como México, donde hacen falta políticas en salud y datos epidemiológicos, para conocer el impacto real de esta enfermedad. El objetivo de este estudio es brindar una visión general de la epidemiología de la epilepsia en México (1970-2020).

**Métodos** Se realizo unas búsquedas bibliografica de estudios epidemiológicos descriptivos sobre epilepsia en regiones rurales y urbanas de México desde 1970 a 2020. Se extrajeron los datos disponibles sobre las características sociodemográficas, prevalencia e incidencia. Finalmente, el resumen, la revisión completa del texto y la extracción de datos se realizaron por duplicado y se informaron utilizando PRISMA. Se utilizo estadística descriptiva.

**Resultados** Se incluyeron 11 estudios epidemiológicos heterogeéneos. En total la prevalencia de epilepsia en México es 3.9-41: 1000 habitantes; 3,9 a 41 por 1000 personas en las regiones rurales y 3,49 a 44,3 por 1000 personas en las regiones urbanas. Ninguno de estos estudios abordó la incidencia de epilepsia. La prevalencia de la epilepsia en México se ha mantenido sin cambios durante las últimas cinco décadas.

**Conclusiones** Nuestros resultados confirman una alta prevalencia de epilepsia en las areas urbanas y rurales de México que se mantiene sin cambios durante las últimas cinco décadas. Todos los estudios incluidos en esta revisión mostraron múltiples limitaciones metodológicas. Se necesitan estudios epidemiológicos nuevos y sólidos para delinear el perfil epidemiológico de la epilepsia en México.

## INTRODUCTION

Epilepsy is the most common major neurological disorder that affects people of all ages. Worldwide, the frequency of epilepsy is estimated to be 69 million people, with a prevalence of active epilepsy at 6.38 per 1,000 inhabitants. 1 Forty-five (65%) out of 69 million people with epilepsy live within rural regions in developing countries, 17 (25%) million are living in the urban areas of these countries, and the rest, 7 (10%) million people, are living in developed countries. 2 About 5 million live in Latin America and the Caribbean, with a median prevalence interval ranging from 15.8 to 17.8 patients per 1,000 inhabitants. 3 Incidence rates are higher in developing countries, especially in Africa and Latin America, where figures can exceed 150 per 100,000 inhabitants. 3 Globally, almost 2.4 million people are diagnosed with epilepsy each year. 4

The prevalence of epilepsy varies from one country to another and even between different areas within the same country due to a lack of access to medical care for reasons related to limited resources, geography, and due to problems with communicating epidemiological data and addressing it appropriately. To this point, < 40% of Latin American countries had at least 1 epidemiological study on epilepsy. In fact, there is sparce epidemiological information available from local health authorities in these countries, and few epidemiological studies have been conducted, which are essential for creating plans and strategies to address the disparities of epilepsy cases at the local level as recommended by The PanAmerican Health Organization (PAHO) as noted in the last report released in 2013. 5

Mexico is a developing country located in Latin America with > 126 million inhabitants (78.8% urban and 21.2% rural); 6 however, like other developing countries, there has been a lack of overview of the epidemiological studies conducted in the last decades which are necessary for the support of public health policies and strategies in reducing the gaps in the number of epilepsy cases, especially in areas with a higher burden of disease.

### Demographic data of Mexico

Mexico is located in North America and covers 1,972,550 km ^2^ , with a density of ∼ 57 people per km. 2 6 According to The National Institute of Statistics and Geography (INEGI) survey 2020 in Mexico, the population of Mexico is 126,014,024 inhabitants, with a total of 78.84% living in urban areas and 21.16% living in rural areas; 48.8% men and 51.2% women. The distribution in percentages of the inhabitants for the age groups is 18.6% (0 to 14 years old), 67.3% (15 to 64 years old) and 14.1% (≥ 65 years old). 6

The aim of our review is to provide an outline of the epidemiological studies of epilepsy in both urban and rural regions of Mexico from 1970 to 2020.

## METHODS

The present review adhered to the methods described in the Preferred Reporting Items for Systematic Reviews and Meta-Analyses (PRISMA) reporting guidelines. 7

### Search strategy

We searched between November and December 2020 for epidemiological studies (from 1970 to 2015) in Spanish or English conducted in Mexico in five databases: PubMed, Scielo, Health Information from Latin America and the Caribbean (LILACS), Medigraphic, and OVID. The mesh terms used were: *epilepsy* , *Mexico* , *Latin* *America* , *epidemiology* , *prevalence* , *incidence* , and *distribution* . The exactitude of the search strategy was validated by cross-verification with the results of previous reviews. Likewise, we reviewed the bibliographic reference lists of the included studies and previous systematic and descriptive reviews on that subject.

### Study selection

Abstracts and titles of all references were screened in duplicate by 2 independent reviewers to identify original population-based studies on the prevalence or incidence of epilepsy in Mexico.

Articles were included if they met the following inclusion criteria: (1) population-based studies that provide a standardized definition of epilepsy having more than one stage of diagnosis and (2) that the main objective of the study is to determine the prevalence and/or incidence of epilepsy in adults and/or children in Mexico without any language restrictions. 3) reported a prevalence or incidence of epilepsy. We excluded descriptive case-series from single centers, review studies, or other type of studies of epilepsy in Mexico. Disagreements pertaining to the inclusion of articles were resolved by consensus or involvement of a third author, as necessary.

### Data extraction

Data abstraction was completed in duplicate by 2 independent reviewers using a standardized data collection form and stored in a Microsoft Excel (Microsoft Corporation, Redmond, WA, USA) sheet. The extracted variables were author, year of publication, sociodemographic characteristics, location, type of population area (urban or rural), type of study, number of patients, prevalence of epilepsy, and age group (only children, only adults, or both). Regarding age, we stratified the cases into 3 large groups for age-specific distribution: 1. Children (preschool and school age); 2. Adults ≥ 18 years old; 3. Any age (children and adults). For the definition of epilepsy, we relied on a common definition that defines epilepsy as ≥ 2 unprovoked seizures > 24 hours apart.

We defined prevalence as the number of existing cases of epilepsy in a population over the total population at a specific point in time. In the present study, we focused on two types of prevalence: the lifetime prevalence of epilepsy (including active and inactive epilepsy/historical cases over time between birth and assessment) and (2) the prevalence of active epilepsy (individuals reporting recent seizures or those currently taking anticonvulsants). The populational study was defined as a study of a group of individuals taken from the general population who shares a common characteristic, such as age, sex, or health condition. 8 Regarding incidence, none of these studies addressed incidence of epilepsy. After reviewing the abstracts of the articles found; 11 studies focused on the prevalence of epilepsy in Mexico were chosen.

### Data synthesis and analysis

We used descriptive statistics to calculate frequencies, percentages, and the rates of both prevalence and incidence of epilepsy in Mexico based on 1,000 inhabitants. This was done using Microsoft Excel (Microsoft Corporation, Redmond, WA, USA) version 2020.

## RESULTS

**Figure 1:**
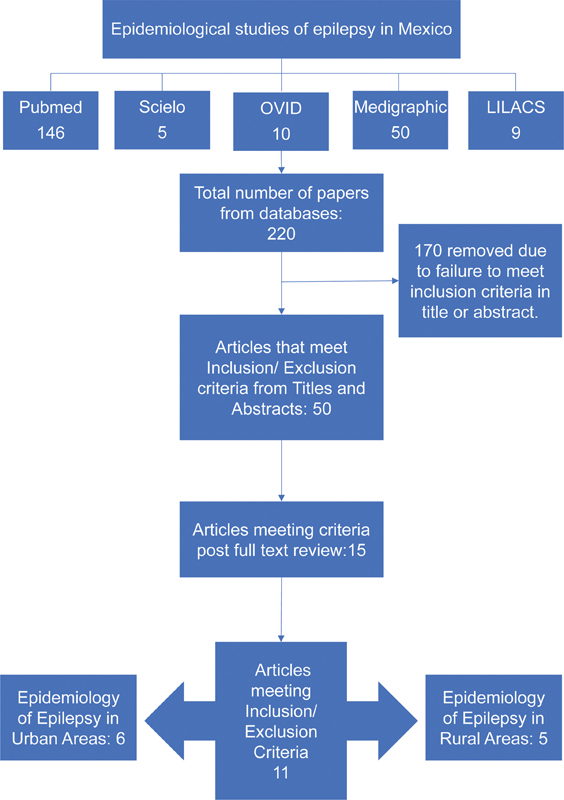
Flowchart of epidemiological studies related to epilepsy in Mexico.

**Figure 2:**
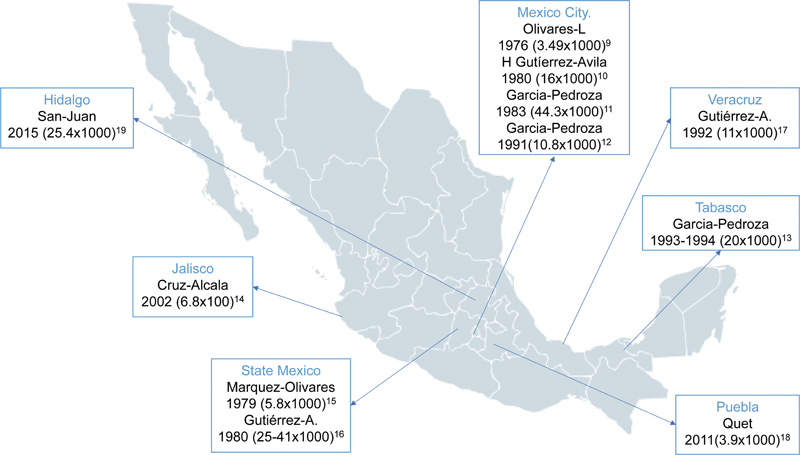
Geographical distribution of Mexico where epilepsy prevalence studies were carried out.

Initially, there were 220 scientific articles recognized, 170 were deemed out-of-scope of the epidemiological study design and were excluded solely based on their abstract. Then, out of the remaining papers, 15 were selected for full text review, and, subsequently, 11 scientific articles met the study criteria and were selected for the present review 9 10 11 12 13 14 15 16 17 18 19 ( Figure 1 ). Tables 1 and 2 show the epidemiological studies according to the rural or urban areas covered. Six (54.4%) studies were conducted in urban areas and 5 (45.6%) studies in rural communities. There was no disagreement among reviewers. The epidemiological studies analyzed were methodologically heterogenous and included few urban and rural regions of Mexico, as shown in Figure 2 .

**Table 1:** Epidemiological studies on the prevalence of epilepsy in urban areas of Mexico (1970-2015)

Author	Study design	Location and setting	Population area	*n*	Prevalence per 1,000 individuals	Age group
Olivares, 1976	Case-control	Mexico City Third level center	Urban	2,169	3.49	Children and adults
Gutíerrez-Avila et al, 1980 10	Cross-sectional study	Mexico City Primary schools	Urban	1,013	16 12.6–23.6	School-aged children
Garcia-Pedroza et al., 1983 11	Cross-sectional study	Tlalpan Mexico City	Urban	1,934	44.3	Children
Garcia-Pedroza, 1991 12	Cross-sectional study	Mexico City	Urban	23,000	10.8	Children
Garcia-Pedroza et al., 1993–1994 13	Door-to-door survey	Comalcalco, Tabasco	Urban	142,000	20	Children and adults
Cruz-Alcala et al., 2002 14	Door-to-door survey	Tepatlitan, Jalisco	Urban	9,082	6.8	Children and adults

**Table 2:** Studies on the prevalence of epilepsy in rural areas of Mexico (1970-2015)

Study	Design	Location	Population Area	*n*	Prevalence per 1,000 individuals	Evaluated group
Márquez et al., 1979 15	Door-to-door survey	Luvianos Tejupilco State of Mexico	Rural	4,103	5.8	Children and adults
Gutiérrez, 1980 16	Door-to-door survey	San Miguel Tecomatlàn State of Mexico	Rural	360	25-41	Children 6–12 years-old
Gutiérrez-A., 1993 17	Door-to-door survey	Nanolinco, Veracruz	Rural	2,025	11	School-aged children
Quet et al., 2011 18	Door-to-door survey	San Andres Azumiatla, Puebla	Rural	4,008/6,203	3.9	Children and adults
San-Juan et al., 2015 19	Door-to-door survey	Xocotitlan, Hidalgo	Rural	863	25.4	Children and adults

In total, the prevalence of epilepsy (urban and rural) was 3.9 to 41:1,000 inhabitants. We found a prevalence of epilepsy ranging from 3.9 to 41 per 1,000 persons in rural regions and 3.49 to 44.3 per 1,000 persons in urban regions of Mexico. None of these studies addressed the incidence of epilepsy. The prevalence of epilepsy in the epidemiological studies analyzed remain unchanged in rural and urban settings during the last 5 decades in Mexico.

## DISCUSSION

The present systematic review found a high prevalence of epilepsy in urban and rural regions in Mexico during the last 5 decades compared with developed countries and globally. The prevalence of epilepsy in the epidemiological studies analyzed remain unchanged in rural and urban settings during the last 5 decades in Mexico. However, there was a significant heterogeneity between the epidemiological studies and caution is needed in interpreting these results. The current etiology of epilepsy in Latin America and Caribbean (LAC continues to be associated with neurocysticercosis (NCC) in 17.3% of the epidemiological studies in this region. Unfortunately, the etiology in Mexican epidemiological studies has not been addressed appropriately; however, local studies show that NCC is considered one of the leading causes of epilepsy. 20

Mexico is a middle-income country, with 62.25 million people living in poverty, which may explain the higher risk of developing epilepsy given that it is reported to a greater degree in the sector of the population considered to occupy a lower socioeconomic status. Furthermore, no epidemiological studies concerning the incidence of epilepsy in Mexico were found. Finally, low levels of education have been shown to translate into low-income levels which are related to poor health conditions, a likely factor for the higher incidence of epilepsy in Mexico. 21

The WHO's 2016 Global Burden of Disease study of epilepsy reported an estimation of 45.9 million patients with all-active epilepsy globally, accounting for 0.56% of total disability-adjusted life-years (DALYs) internationally. The study also found a greater severity and higher sum of years of life lost for premature mortality and years lived with disability due to epilepsy in low-income settings. 22 The prevalence of epilepsy worldwide differs significantly among countries depending on the sociodemographic characteristics, as well as both the local distribution of risk and etiologic factors. The overall global prevalence of epilepsy was 7.60 per 1,000 inhabitants (95% confidence interval [CI]: 6.17–9.38). 23 According to a recent systematic review and meta-analysis of population-based studies of the epidemiology of epilepsy in LAC, there is a lifetime prevalence that ranges from 11.7 to 16.6 patients per 1,000 inhabitants with an incidence range of 111.24 per 100,000 person-years (95%CI: 64.88–169.51). 24 Our results are included in this data. Currently, Mexico is considered one of the countries with the highest prevalence of epilepsy (25:1,000) in Latin America, followed by Chile (17.75:1,000) and Guatemala (12.95:1,000); however, this estimation was established based on only 4 epidemiological studies from Mexico. 24 Interestingly, in the present study, there were no differences were observed in relation to setting, whether it be urban/rural, or differences attributable to sex or age clusters, or even the income in LAC.

A recent report of the Prevention Task Force of the International League of Epilepsy conducted a systematic review of published epidemiologic studies of epilepsy of 4 preventable etiologic categories: perinatal insults, traumatic brain injury (TBI), CNS infection, and stroke. In fact, CNS infections were a more common attributed cause in low- and middle-income countries (LMIC), accounting for ∼ 5% of all epilepsy cases. Among some rural LMIC communities, the median proportion of epilepsy cases attributable to endemic NCC was as high as 34%. Therefore, a large proportion of the overall public health burden of epilepsy is due to preventable causes as well as to the attributable fraction for perinatal causes, infections, TBI and stroke, in sum reaching nearly 25%. 25

Significant limitations of the epidemiological studies used for the present review include the low sample size of each study, the heterogeneity of the studies, insufficient detail in describing the epilepsy types, the absence of stating epilepsy status (active or resolved), and the lack of mention of etiological sources.

Worthy of attention are the results of our study that show that the prevalence of epilepsy in Mexico in both rural and urban settings remain unchanged during the last 5 decades, even with scientific and technological advances in the sector of corresponding health issues; all the while, there continues to be a lack of access to health services in developing countries, and even more, in rural communities which greatly limit the diagnosis and treatment of epilepsy. This causes the prevalence to be higher in these regions compared with developed countries or communities with greater access to health services. 26 In such a way that epilepsy is a public health problem that must be taken seriously, public policies must reflect this and be generated in order to guarantee access to adequate health services.

Furthermore, new, robust, and broad prospective epidemiological studies are needed to determine the national epidemiological profile of patients with epilepsy in Mexico to contribute delineated health policies, research investment and dedicated health infrastructure to reduce the existing gap disparities in epilepsy.

In conclusion, our results confirm a high prevalence of epilepsy in both urban and rural settings in Mexico that remain unchanged during the last 5 decades. All studies included in the present review showed multiple methodological limitations. The heterogeneity of the results could be due to differences in the population samples, the use of differing screening methods, the definitions and classifications used in the questionnaires, and selection bias. Caution is needed to interpret our findings. New and robust epidemiological studies are needed to delineate the epidemiological profile of epilepsy in Mexico.
